# Research priorities for rare neurological diseases: a representative view of patient representatives and healthcare professionals from the European Reference Network for Rare Neurological Diseases

**DOI:** 10.1186/s13023-020-01641-z

**Published:** 2021-03-18

**Authors:** Annemarie E. M. Post, Thomas Klockgether, G. Bernhard Landwehrmeyer, Massimo Pandolfo, Astri Arnesen, Carola Reinhard, Holm Graessner

**Affiliations:** 1 Institute for Medical Genetics and Applied Genomics, University of Tübingen, Tübingen, Germany; 2 Centre for Rare Diseases, University Hospital Tübingen, Tübingen, Germany; 3 Department of Neurology, University of Bonn, Bonn, Germany; 4 German Center for Neurodegenerative Diseases (DZNE), Bonn, Germany; 5 Department of Neurology, University of Ulm, Ulm, Germany; 6 Service of Neurology, Erasme Hospital, and Laboratory of Experimental Neurology, Université Libre de Bruxelles, Brussels, Belgium; 7 European Huntington Association (President), Søgne, Norway

**Keywords:** Rare diseases, Patient involvement, Research themes

## Abstract

**Background:**

Patient involvement in research increases the impact of research and the likelihood of adoption in clinical practice. A first step is to know which research themes are important for patients. We distributed a survey on research priorities to ERN-RND members, both patient representatives and healthcare professionals, asking them to prioritize five research themes for rare neurological diseases on a scale ranging from 1 (most important) to 5 (least important). A follow-up e-mail interview was conducted with patient representatives and professionals to assess potential reasons for differences in opinions between these two groups.

**Results:**

In total, 156 responses were analysed: 61 from professionals and 95 from patient representatives. They covered all ERN-RND disease groups and came from 20 different EU countries. Almost half of the respondents considered ‘Developing therapies and preventive strategies’ the most important research theme. In particular, patient representatives prioritized this theme more often than professionals, while professionals prioritized ‘Disease mechanisms and models’. Patient representatives indicated that therapies and prevention were of the utmost importance to them, because their lives are often heavily impacted by the disease and their main goal is to relief the burden of disease. Professionals indicated that investigating disease mechanisms will lead to more knowledge and is indispensable for finding new treatments.

**Conclusions:**

Patients and professionals have different opinions on which research theme should have priority. A qualitative follow-up shows that they respect each others’ view points. Different stakeholders involved in research should be aware of their differences in research theme priority. Explaining these differences to each other leads to more understanding, and could improve patient engagement in research.

**Graphical Abstract:**

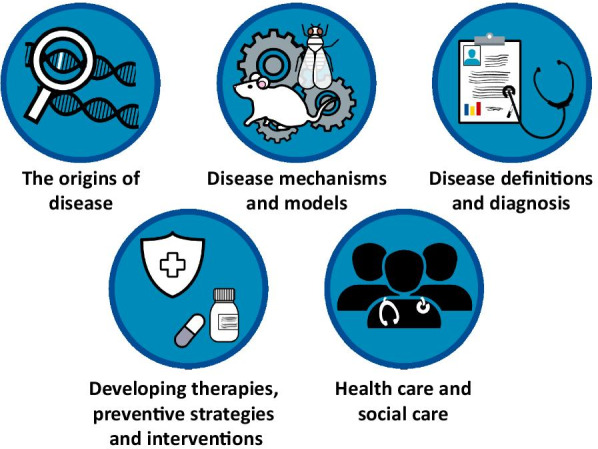

## Introduction

### Rare diseases

In Europe, a disease is classified as ‘rare’ if it has a prevalence of no more than five affected persons per ten thousand [1]. However, the more than seven thousand known rare diseases have a considerable collective impact, with more than thirty million people estimated to be affected in the European Union alone [2]. Because of the low prevalence of each distinct rare disease entity, the overall large total number of patients, and the heterogeneity of rare diseases, research on rare diseases requires concerted action at the European level, according to EURORDIS [3]. Moreover, this research needs to be multidisciplinary, patient-centred, and sustainable on the long-term [3].

### ERN-RND

European Reference Networks (ERNs) have been established to meet these research requirements. ERNs were launched in 2017 as a result of the adoption of Directive 2011/24/EU [4]. Currently, there are 24 such virtual networks, involving over three hundred hospitals in 25 EU countries. In addition to promoting research, their goal is to share the highly specialized knowledge needed to care for people affected by rare diseases, so that all patients in need can be reached. According to IRDiRC, ERNs provide a unique opportunity to improve standards of care and to increase access to diagnosis and treatment for patients [5].

The ERN for Rare Neurological Diseases (ERN-RND) focuses on the following disease groups:

Cerebellar Ataxias & Hereditary Spastic Paraplegias; Huntington’s disease and other Choreas; Frontotemporal dementia; Dystonia, (non-epileptic) Paroxysmal Disorders and Neurodegeneration with Brain Iron Accumulation; Atypical Parkinsonian Syndromes; and Leukodystrophies.

### Research priorities for rare diseases

Research priorities for rare diseases are defined by the challenges that patients face, mainly to obtain an accurate and timely diagnosis and to have appropriate treatments available [5]. Although the diagnosis of rare disease was revolutionized during the last decade by the introduction of next‐generation genomics and the concomitant discovery of many new disease-causing mutations in novel genes, many patients experience delays in receiving a correct diagnosis or do not obtain a diagnosis at all [6]. As for therapies, despite some progress [7], no effective treatment is available for approximately 95% of rare diseases [5]. Moreover, the yearly number of newly approved treatments for previously untreatable rare diseases remains low [5]. Progress in rare disease diagnosis and treatment depends on a multitude of factors. Better insight into pathological mechanisms is essential to identify therapeutic targets, which may be shared by multiple diseases involving the same molecular pathway [8], as well as to allow the development of diagnostic tests and biomarkers.

### The role of patients in setting research priorities

In its position paper [3], EURORDIS states the centrality of patients in research, who are its ultimate beneficiaries, supporting a patient-centred approach to research projects, the active participation of patients, and the sharing of results with patients [3]. Others also pointed out that research efforts that are relevant to patients should be prioritized [9]. Therefore, projects need to address relevant clinical questions and focus on patient-centred health outcomes [10]. Moreover, involving patients increases the impact of research, as well as the likelihood that its outcomes will be adopted in clinical practice [9, 11]. In its position paper on patient involvement in neuroscience research, the European Federation of Neurological Associations (now European Academy of Neurology) sets as a priority that action should be taken ‘to ensure that the research community understands what is important to people with health conditions’ [12].

### Aim of this survey

We collected the opinion of patient representatives and healthcare professionals within ERN-RND on the prioritization of research priorities for rare neurological diseases. For this purpose, we used the main research themes that the EU Joint Programming for Neurodegenerative Disease Research (JPND) has identified in its strategic research agenda [13]. These research themes include: The origins of disease; Disease definitions and diagnosis; Developing therapies, preventive strategies and interventions; Disease mechanisms and models; Healthcare and social care.

## Results

One hundred fifty-six responses to the survey regarding research priorities were collected and analysed. The number of times a certain theme was prioritized as ‘most important’ was counted. Out of the 156 respondents, 74 prioritized ‘Developing therapies and preventive strategies’ as most important (47%, Table 1). The theme ‘Disease mechanisms and models’ was considered the least important, as only 12 respondents prioritized it (8%, Table 1).

**Table 1:** Number and percentage of respondents that prioritized each theme as most important

	*n*	%
**Total**	**156**	**100**
The origins of disease	33	21.2
Disease definitions and diagnosis	18	11.5
Developing therapies and preventive strategies	74	47.4
Disease mechanisms and models	12	7.7
Healthcare and social care	19	12.2

We investigated differences in subgroups of respondents, namely patient representatives versus healthcare professionals, and advanced- versus early-stage professionals.

### Patient representatives versus healthcare professionals

**Fig. 1:**
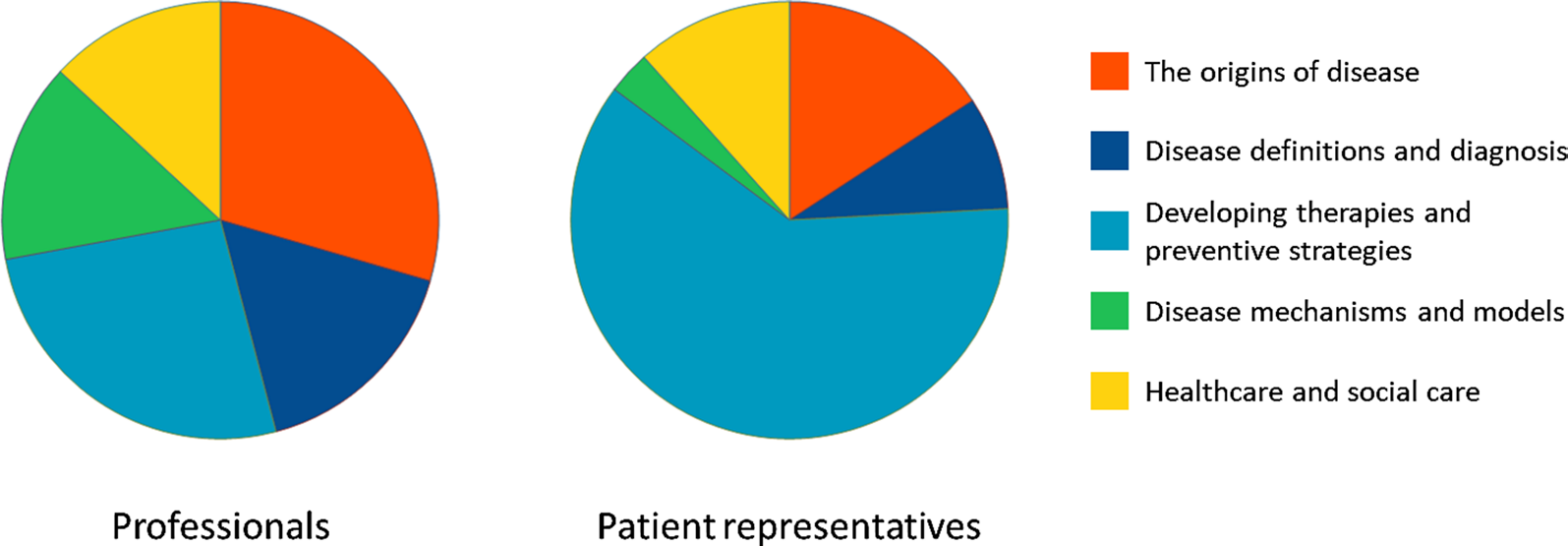
Prioritization of research themes by professionals and patient representatives

We compared the prioritizations of 95 patient representatives with those of 61 healthcare professionals. Sixty-one percent of the patient representatives prioritized ‘Developing therapies and preventive strategies’, while only 26% of the professionals made the same choice (Table 2 and Fig. 1), making this theme the second-most important for professionals. Thirty percent of the professionals ranked ‘The origins of disease’ as most important, while only 16% of patient representatives prioritized this theme (Table 2 and Fig. 1), which was ranked as second-most important by patient representatives.

**Table 2:** Number and percentage of respondents that prioritized each theme as most important, divided in professionals and patient representatives

	Professionals		Patient representatives	
	*n*	%	*n*	%
**Total**	**61**	**100**	**95**	**100**
The origins of disease	18	29.5	15	15.8
Disease definitions and diagnosis	10	16.4	8	8.4
Developing therapies and preventive strategies	16	26.2	58	61.1
Disease mechanisms and models	9	14.8	3	3.2
Healthcare and social care	8	13.1	11	11.6

In order to further investigate the differences between patient representatives and professionals, we focused on the two themes showing the largest difference in prioritization: ‘Developing therapies and preventive strategies’, prioritized by more than twice as many patient representatives compared to professionals, and ‘Disease mechanisms and models’, rated as most important by 4.7 times more professionals than patient representatives. We asked patient representatives who rated ‘Developing therapies and preventive strategies’ as most important and professionals who rated ‘Disease mechanisms and models’ as most important for their reasons for making this choice (Table 3).

**Table 3:** Answers from six patients to the interview questions

**Why did you choose the theme 'Developing therapies and preventive strategies' as most important?**	
It evokes in me the hope of anticipating and controlling the evolution of the disease.	
I believe that, regardless of the situation and personal involvement, it is essential that everyone is committed and involved in research in order to achieve a better quality of life.	
For me medical research is very important	
(1) to be able to develop strategies to help to diagnose a patient as early as possible in order to avoid any further psychological problems posed by diagnostic delay. It is very important too that a diagnosis be posed as early as possible in order to offer preventive strategies to enable the patient to continue his/her lifestyle as long as possible.	
(2) In the long term to develop therapies to help slow or even cure the disease.	
These two aspects have priority for me, as a therapy could alleviate or slow down the progression of the disease. In addition, one could prevent a possible manifestation in children who have inherited the defective gene. With such a possibility one would certainly be able to reduce the psychological problems of the patients.	
It feels like it would be the most impactful area of research for me in terms of having the potential to change the outcome of how the disease would impact me personally (managing/preventing it).	
To the extent that, to date, it is still not possible to reverse or "repair" neuronal lesions, and that we have elements to know the potential for future patients, this seems to me to be an avenue to consider.	
**What is your opinion on the theme 'Disease mechanisms and models'?**	
It is not very clear to me.	
I think it depends a lot on the type of illness. In some cases studying mechanisms and trying to make "models" is useful not only for researchers, but also for doctors who have to recognise the disease or exclude it. I think, therefore, that it is very important to study it as much as possible in order to understand it and deal with it in the best way.	
I do find that it is important to do research on how the disease appears and spreads, why in some patient very slowly and in another faster. I think it's very important to know what's happening in order to develop efficient medicine.	
In order to understand a disease and its consequences, one must first know how it develops and which changes in the body occur and what the consequences are. Since the predisposition for Huntington's disease is already present in the genome, it is necessary to find out why some people have "normal" gene sequences and some have a high or very high number of corresponding gene sequences. Since genetic research is advancing all the time, we will certainly find a way to modify the corresponding genes in the near future.	
It seems fundamental as a basis for knowledge and understanding and therefore for being able to move on to therapies and prevention with this knowledge. However when you are faced with a disease it feels less important on an individual level. I can definitely see the overall importance of this research area though.	
It is a theme that should be able to improve and deepen even more the knowledge on pathologies and which can lead to preventive and/or therapeutic solutions.	
**Why is the research theme 'Developing therapies and preventive strategies' more important for you than the theme 'Disease mechanisms and models'?**	
In my opinion, the priority in the field of research would be to put in place the means to slow down the progression of the disease; to maintain autonomy.	
I understand the importance (of the theme ‘Disease mechanisms and models’) as a patient I do have a lot of frustration towards that as I know we are talking here of a very, very long time (several decades). So I think it would be wise to develop preventive therapies that a patient can do and experience and perhaps to let the patients know what's happening in the long term research from time to time in some ways.	
I can't answer that for sure. Maybe I unconsciously gave this answer for selfish reasons. My husband has Huntington's disease, but we don't have children. An effective therapy would be advantageous for me, I would not lose him and I would not have to watch how he decays, physically and mentally.	
I think that both are important aspects, from the point of view of the patient or the care giver, especially where there is a genetic implication, it is very important to be able to give "hope". Perhaps prevention also makes it possible to slow down the course of the disease and to study in greater depth the dynamics of its development.	
The damage caused is usually irreversible. Anything that can be done before the symptoms appear is of the utmost importance.	
**Can you imagine that clinicians think it is important to do research on 'Disease mechanisms and models'?**	
I think this is useful in order to be able to recognise the disease as early as possible and thus to obtain a quick diagnosis with greater possibilities of treatment.	
While I can understand the importance of this area to clinicians, it is not as obvious to me personally what this fully involves and therefore the immediate value.	
Yes I can understand that the clinicians do that and even I would be very suspicious on the scientific value of the outcome of a research where they would not do so. I think generally that the medicine cannot come up with a cure to a disease if they don't investigate the disease mechanisms. This being said, as I think it will take many years, I do understand the frustration among patients.	
Almost all of us are waiting for results, for solutions so that patients can at least live better with their disease. All areas of research are important, let's trust the researchers, many of whom are listening to patients and their families, and that this listening should probably guide their thinking to some extent.	
**What needs to be done so that clinicians and patients understand each other's needs in which research theme needs to have priority?**	
I think it's important for family and doctors to meet. It would be appropriate that on the occasion of large events such as workshops and dedicated congresses, family members' associations can participate, perhaps with meetings "on the side". So that they can express their needs. Also from the point of view of methods of communication and approach to diagnosis.	
Conversation! Patients having a rationale and understanding of the gains to be had from the priority areas of clinicians. Clinicians are looking at an overall picture and long term gains, whereas they need to understand that a patient isn’t usually coming at it from a selfless perspective-they usually want what will be best for themselves within their life span.	
I would say a lot more interactions between them, a lot more information and links between the patients and the clinicians, for example through the patients associations.	
Personally, I joined an association as soon as I was diagnosed. I found there a listening ear, information, exchanges between people "concerned" to varying degrees. I also had the opportunity to exchange with research project leaders or researchers. This bilateral consideration is considerably appreciable because it allows me to become aware that these are not two distinct worlds but two intertwined worlds, one for the other, one with the other.	

Patient representatives answered that having control over their disease was essential to reduce the impact of the disease on their life (Table 3). Their view on the theme ‘Disease mechanisms and models’ were in general favourable as well, they agreed that such research is necessary and sympathized with the view of professionals that it is an important research theme, but pointed out the more direct impact of research into ‘Developing therapies and preventive strategies’ (Table 3).

Professionals thought it critical to do research on ‘Disease mechanisms and models’, because they see it as a crucial step on the way to ‘Developing therapies and preventive strategies’ as the ultimate goal (Table 4). When asked what they thought was the motivation for patient representatives to directly prioritise ‘Developing therapies and preventive strategies’, they responded that it is important for patients to be able to live their life as normal as their stage of the disease permits.

**Table 4:** Answers from four professionals to the interview questions

**Why did you choose the theme 'Disease mechanisms and models' as most important?**	
All the themes listed are extremely important in the research field. With my answer I intended to highlight how central the role of disease mechanism comprehension should be in the road to therapy development, preventive strategies, healthcare and disease diagnosis. In other words, I believe that all the others are not truly possible without this nuclear field.	
I mentioned this point as it is a sub heading of origins of disease in my mind. But often 'origins of disease' is mainly genetic diagnosis or genetic research, as we more and more see that genetic tests give an answer in only a small proportion of dystonias. Therefore it is important to tackle the underlying mechanisms of the disease in terms of networks and models of these networks. This may open new insight into the origin of disease and new therapeutic models.	
I consider the lack of good disease models that validly represent the disease in humans and the lack of mechanistic understanding to be the biggest bottleneck on the way to a causal therapy. Developing therapies for me is inherently linked to this. Everything else is secondary for me because it is not causal or 'disease-modifying'.	
Based on these research findings, we can develop new treatments. It allows us to see the disease in a broader perspective. We first need to understand the mechanism and then think about how to treat the disease.	
**Can you imagine that patients think it is most important to do research on 'Developing therapies and preventive strategies'?**	
Yes. Patients are very disabled by the disease, socially and physically. This is both a burden and a stigma. We try our best to help them, but the results may not be good enough for them to go back to a normal life. Therefore they hope to get a better cure and preventive treatments to get back to normal or the best condition possible.	
This is a huge topic, particularly from the patients' point of view. But also from ours: this is the final goal shared between all of us. Research on this topic is very important, but unfortunately not possible yet for all diseases. We still miss important information about disease mechanisms. This is the crucial point. However, studying disease mechanisms (and models) is the first step for developing therapies.	
I can very well imagine that this is the most important issue for patients. We need causally effective "disease-modifying therapies". Patients see it as hope from the end, I as a scientist from the necessary beginning and stony path (disease models and mechanism).	
Yes. Patients want to have a treatment in their lifetime.	
**What needs to be done so that clinicians and patients understand each other's needs in which research theme needs to have priority?**	
We already communicate on a regular basis with patient's associations. If we want to broaden our view, we could: • Increase the amount of webinars and (when possible) meetings with patient's associations. • Ask patients to complete a survey about research. How do they see and understand it; what are the different sources where they can find it; to what degree do they understand the objective, results and relevance of research; do they understand the differences between clinical/therapeutic and pathophysiology? • Propose research objectives or projects on the websites of patient organisations. This will Increase their awareness and understanding. It may motivate them to be involved in research (which is currently not at its optimal rate). • Make the results of this survey available on the website of ERN-RND. • Organize an (online) discussion on this topic.	
I had really good experiences regarding 'mixed' meetings: with scientists and families. I found the families' part of these meetings very interactive and useful for both sides. Sometimes researchers need to remind themselves what the disease burden is for patients and families.	
It is crucial that patients understand that the development of a drug requires understanding of mechanisms, and the transfer tot the clinic the necessary intermediate step of testing in good models—both of which require patience. I believe that every doctor-patient contact should contribute to this exchange of knowledge. Institutionally, I see the opportunity for strong involvement of patient organizations in scientific congresses and on the other hand the invitation of professionals to events of the patient organizations.	
There needs to be collaboration between patients and researchers. In my experience there is no need to convince patients about the need for research on disease mechanisms. They do have a different perspective. But for example in research studies, patients ask 'will this help me?', and when we explain that unfortunately it will not, but it may help the next generation of patients, they think this is a good reason to participate.	

The two groups were asked what needs to be done so that professionals and patients understand each other’s needs in prioritising research themes. Both emphasized the importance of dialog. The professionals thought it important for patients to understand why research into disease mechanisms is crucial for the development of new therapies. Patients thought it important for professionals to understand the impact of the disease on their daily life.

### Advanced- versus early-stage professionals

**Fig. 2:**
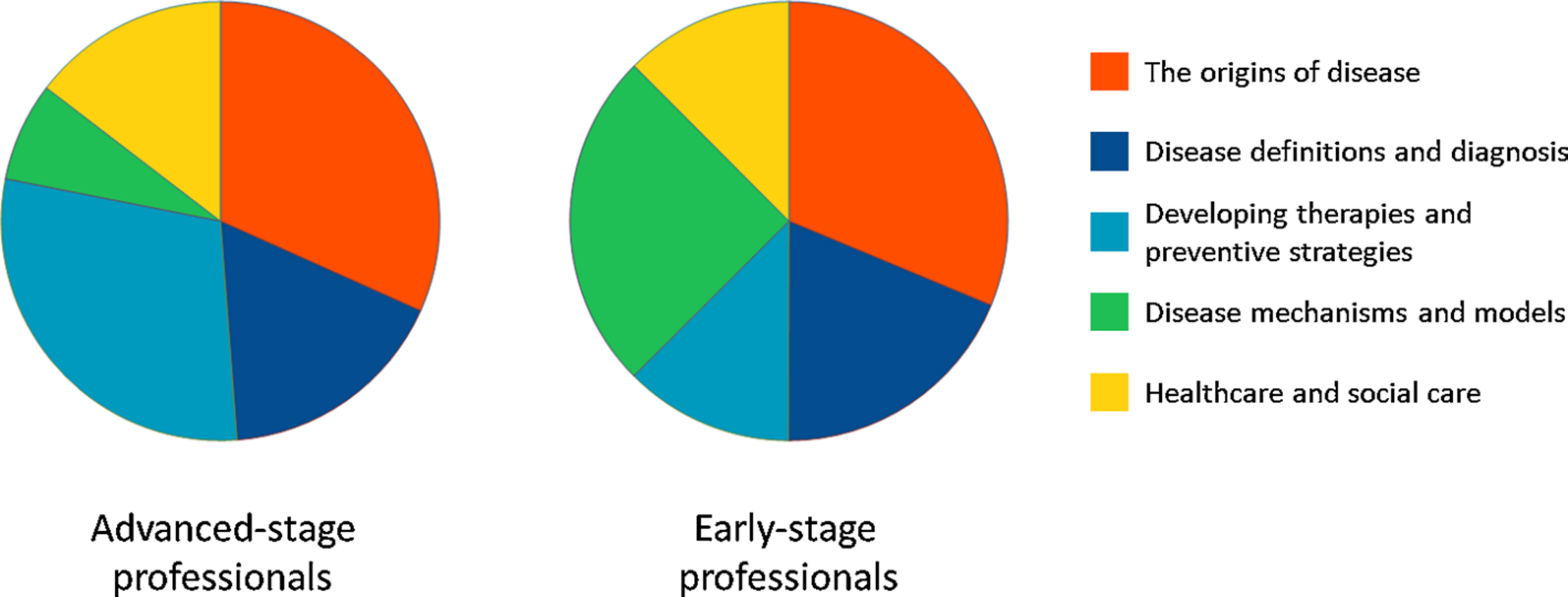
Prioritization of research themes by advanced- and early-stage professionals

The prioritizations of professionals who had reached a career level corresponding to “attending physician” or “laboratory director” were compared to those of professionals who had not yet reached such career level. Forty-one ‘advanced’-stage professionals and 16 ‘early’-stage professionals responded. Four professionals who were experts other than clinicians or researchers were excluded. Both advanced-stage and early-stage professionals prioritized ‘The origins of disease’ as most important, but the groups differed in their prioritisation of the themes ‘Developing therapies and preventive strategies’, chosen by 29% of the advanced-stage professionals compared to 13% of the early-stage professionals (Table 5 and Fig. 2), and ‘Disease mechanisms and models’, prioritized by 25% of early-stage professionals compared to 7% of advanced-stage professionals.

**Table 5:** Number and percentage of respondents that prioritized each theme as most important, divided in advanced-stage and early-stage professionals

	Advanced-stage		Early-stage	
	*n*	%	*n*	%
**Total**	**41**	**100**	**16**	**100**
The origins of disease	13	31.7	5	31.3
Disease definitions and diagnosis	7	17.1	3	18.8
Developing therapies and preventive strategies	12	29.3	2	12.5
Disease mechanisms and models	3	7.3	4	25.0
Healthcare and social care	6	14.6	2	12.5

We did not pursue a qualitative analysis of the opinions of these groups due to a rather low response rate when asked to participate in a follow-up interview.

## Discussion

Here, we present the results of a survey on research priorities of ERN-RND members, both patient representatives and healthcare professionals. Almost half of the respondents considered ‘Developing therapies and preventive strategies’ the most important research theme, more so than ‘The origins of disease’, ‘Disease definitions and diagnosis’, ‘Disease mechanisms and models’, or ‘Healthcare and social care’.

Patient representatives prioritized ‘Developing therapies and preventive strategies’ more often than professionals, while more professionals prioritized ‘Disease mechanisms and models’. An e-mail interview was conducted with patient representatives and professionals to assess potential reasons for this difference. Patient representatives indicated that therapies and prevention were of the utmost importance to them, because their lives are often heavily impacted by the disease and their main goal is to relief the burden of disease. Professionals indicated that investigating disease mechanisms will lead to more knowledge and is indispensable for finding new treatments.

Patient representatives and professionals both agreed that a dialogue between patients and professionals is essential to understand each other’s views and expectations. Patient representatives suggested several options for such interactions. The patients’ voice and opinions are brought in by European patient advocacy groups (ePAGs, managed by EURORDIS), which are active in all ERN-RND disease and working groups and are represented in all decision making bodies.

Within ERN-RND, a discussion on research priorities between patient representatives and professionals can be included in the Disease Groups. More in general, EURORDIS and EFNA (European Academy of Neurology), among others, provide training and information about different processes in medical research to enable patient representatives to understand how research is being performed. A good example of such training courses are Schools organised by the EURORDIS Open Academy (https://openacademy.eurordis.org/).

In addition, there were differences in prioritization between advanced-stage and early-stage professionals. The former more often chose ‘Developing treatments and preventive strategies’ as most important, while the latter deemed ‘Disease mechanisms and models’ to be more important. Due to the limited number of responses, we cannot give the reasons for this difference here. Possibly early-stage professionals, who perform ‘hands-on’ research, are more directly involved in studying disease mechanisms and developing models to learn as much as they can about the disease they are specializing in. On the other hand, advanced-stage professionals may feel more compelled to directly address patient needs.

In this study we did not assess differences in prioritization of research themes between clinical and laboratory-based professionals. Most professionals that replied to the survey indicated that they were clinicians (n = 34), while others identified themselves as both clinician and laboratory-based scientist (n = 22). Only one respondent indicated that they were solely laboratory-based, which made an analysis of differences between these two groups impossible.

Moreover, we did not assess differences in research theme prioritization between different countries. Of the 20 countries from which we received responses to our survey, only two countries (Germany and France) were more than 20 times represented. Moreover, the amount of patient representatives and professionals per country is very skewed. From some countries, we only received replies from patient representatives (Australia, Austria, Ireland, Malta, Norway, Sweden, and Switzerland), while from other countries only professionals replied to our survey (Bulgaria, Czech Republic, Hungary, Lithuania, Slovenia, and Spain).

There are various methods that can be used for priority setting of research themes, amongst others the health equity lens model, and the James Lind Alliance (JLA) priority setting partnership model. In these models, patient representatives are already involved in the formulation of the research themes. In the health equity lens model, a survey is then conducted among a different set of patients to prioritize these research themes [14], while the JLA process makes use of workshops with patients for the same purpose [11]. The five JPND research priorities used in this analysis were developed in collaboration with various stakeholders, among which were patient representatives [13].

Patient engagement in research does not end with priority setting. There are multiple other ways in which patients can be involved in research, for example in the planning and conduct of research in particular on outcome measure that reflect real life [10]. In the view of ERN-RND ePAGs, patients are increasingly involved in the preparation and development of protocols for clinical trials, as well as the results from trials being shared with patients. Moreover, there are several patient-initiated projects, of which HEALTHE-RND is an example. In this project a unique disease-specific quality of life instrument is developed, which is not symptom-based, but focuses on enabling the patient.

## Conclusions

Patients representatives and professionals have different opinions on which research theme should have priority. A qualitative follow-up shows that they respect each others’ view points. Different stakeholders involved in research should be aware of their differences in research theme priority. Explaining these differences to each other leads to more understanding, and could improve patient engagement in research.

## Methods

All information regarding the ERN-RND can be found on http://www.ern-rnd.eu.

The survey on research priorities was designed by the ERN-RND working group on research and registries, in particular Thomas Klockgether, G. Bernhard Landwehrmeyer, Massimo Pandolfo, and Holm Graessner. Initially, the survey was sent to all ERN-RND participants including patient representatives in January 2018 (Additional file 1). The question posed by the survey was to prioritize five research themes for rare neurological diseases on a scale with five levels, ranging from 1 (most important) to 5 (least important). In addition, the respondents were asked to indicate in which country they were based, what their main disease group focus was, and, if applicable, their profession and career position. The survey was conducted in English. Seventy-two individuals responded, including eight patient representatives and 64 healthcare professionals from a total of 16 countries, covering all ERN-RND disease groups (Additional file 2). Three answers from professionals were excluded from the analysis, because the respondents sent multiple replies to the survey, in which the prioritization of research themes was inconsequent.

In January 2020, additional patient representatives were approached and asked to fill out the survey, in order to improve the balance between responses from patient representatives and professionals. An additional item was included inquiring whether participants agreed to receive follow-up e-mails with regards to this survey. This additional survey was conducted in seven languages: English, German, French, Italian, Dutch, Polish, and Czech. Three hundred and two responses were received, but only 87 were included in the analysis after excluding answers from professionals (n = 5), and answers that used the same rank for multiple research themes (n = 201). The 87 analysed responses originated from 14 different countries. They covered five of the six disease groups of ERN-RND; Leukodystrophies were not included (Additional file 2). This brought the total number of responses analysed to 156, 61 from professionals and 95 from patient representatives. In total, responses from 20 different countries were included in the analysis (Additional file 2).

Twenty-two patient representatives and nine professionals were asked to comment on why they had prioritized a certain theme as most important. These subgroups were chosen based on differences in prioritization of the research themes, and asked for their reasons to prioritize a certain research team. Respondents were able to opt out of their participation at any time without having to provide a reason, and all responses were anonymised. We received answers from six patient representatives and four professionals.

## Supplementary Information

**Additional file 1**. The survey that was distributed to ERN-RND members and patients representatives.

**Additional file 2**. The country and disease group focus of the survey’s respondents.

## Data Availability

Not applicable.
