# Supplementary material for: Research priorities for rare neurological diseases: a representative view of patient representatives and healthcare professionals from the European Reference Network for Rare Neurological Diseases
Source: Orphanet J Rare Dis. 2021 Mar 18;16:135. doi: 10.1186/s13023-020-01641-z (PMC7976714; doi:10.1186/s13023-020-01641-z)
Supplement: Supplementary file 1 — Additional file 1. The survey that was distributed to ERN-RND members and patients representatives. [file 13023_2020_1641_MOESM1_ESM.pdf]

Bio data

Country

……

Main disease group focus

- Ataxia / HSP
- Atypical parkinson's disease
- Huntington's disease and choreas
- Dystonia / NBIA / Paroxysmal disorders
- Frontotemporal dementia
- Leukodystrophies

Profession and career position (healthcare professionals)*

Clinicians*

- At least consultant level
- Not reached consultant level

Laboratory based scientists*

- At least laboratory manager level
- Not reached laboratory manager level

Other*

- Yes
- No

Patient / patient representative*

- Yes
- No

Prioritisation of research themes for rare neurological diseases

Please RANK the following research themes regarding their relevance for the activities of the ERN-RND during the next 5 years by ordering the 5 topics/items listed below in order of priority (1-most relevant, 5-less relevant)
(*Please do NOT assign an identical rank score to items of similar importance, but rank-order all items*)

- The origins of disease
- Disease definition and diagnosis
- Developing therapies, preventive strategies
- Disease mechanisms and models
- Healthcare and social care

* only in the survey of January 2018
